# Early and annual projected savings from anti-CGRP monoclonal antibodies in migraine prevention: a cost-benefit analysis in the working-age population

**DOI:** 10.1186/s10194-024-01727-0

**Published:** 2024-02-12

**Authors:** Carlos Lazaro-Hernandez, Edoardo Caronna, Joana Rosell-Mirmi, Victor J Gallardo, Alicia Alpuente, Marta Torres-Ferrus, Patricia Pozo-Rosich

**Affiliations:** 1 Neurology Department, Vall d’Hebron University Hospital, Barcelona, Spain; 2 Headache Clinic, Neurology Department, Vall d’Hebron University Hospital, Barcelona, Spain; 3 Headache and Neurological Pain Research Group, Vall d’Hebron Research Institute, Department of Medicine, Universitat Autònoma de Barcelona, Barcelona, Spain

## Abstract

**Background:**

Migraine is one of the main causes of disability worldwide. Anti-CGRP monoclonal antibodies (MAbs) have proven to be safe and efficacious as preventive migraine treatments. However, their use is restricted in many countries due to their apparently high cost. Cost-benefit studies are needed.

**Objective:**

To study the cost-benefit of anti-CGRP MAbs in working-age patients with migraine.

**Methods:**

This is a prospective cohort study of consecutive migraine patients treated with anti-CGRP MAbs (erenumab, fremanezumab and galcanezumab) following National reimbursement policy in a specialized headache clinic. Migraine characteristics and the work impact scale (WPAI) were compared between baseline (M0) and after 3 (M3) and 6 months (M6) of treatment. Using WPAI and the municipal average hourly wage, we calculated indirect costs (absenteeism and presenteeism) at each time point. Direct costs (emergency visits, acute medication use) were also analysed. A cost-benefit study was performed considering the different costs and savings of treating with MAbs. Based on these data an annual projection was conducted.

**Results:**

From 256 treated working-age patients, 148 were employed (89.2% women; mean age 48.0 ± 8.5 years), of which 41.2% (61/148) were responders (> 50% reduction in monthly headache days (MHD)). Statistically significant reductions between M0 and M3/M6 were found in absenteeism (*p* < 0.001) and presenteeism (*p* < 0.001). Average savings in indirect costs per patient at M3 were absenteeism 105.4 euros/month and presenteeism 394.3 euros/month, similar for M6. Considering the monthly cost of anti-CGRP MAbs, the cost-benefit analysis showed savings of 159.8 euros per patient at M3, with an annual projected savings of 639.2 euros/patient. Both responders and partial responders (30–50% reduction in MHD) presented a positive cost-benefit balance. The overall savings of the cohort at M3/M6 compensated the negative cost-benefit balance for non-responders (< 30% reduction in MHD).

**Conclusion:**

Anti-CGRP MAbs have a positive impact in the workforce significantly reducing absenteeism and presenteeism. In Spain, this benefit overcomes the expenses derived from their use already at 3 months and is potentially sustainable at longer term; also in patients who are only partial responders, prompting reconsideration of current reimbursement criteria and motivating the extension of similar cost-benefit studies in other countries.

**Supplementary Information:**

The online version contains supplementary material available at 10.1186/s10194-024-01727-0.

## Background

Migraine is a highly prevalent disease which peaks during the most professionally productive years of our lives [1]. According to the last report of the Global Burden of Disease (GBD) study, headache rates as the second most disabling disease in terms of disability-adjusted life years (DALYs) in people under the age of 50 [1, 2] and, especially, in Western Europe [2]. It represents the 5% of the DALYs between 10 and 24 years of age and 3.7% between 25 and 49 years [1]. This leads to huge direct and indirect costs for the society reaching the 111 billion annually in Europe [3, 4]. Indirect costs (absenteeism and presenteeism) account for the biggest part of this economic burden [3], making the actively working population a relevant target for migraine preventive strategies.

Oral preventive drugs (such as beta-blockers, antiepileptics and antidepressants) are the most widely available and used treatments for migraine prevention. However, they are non-specific and often associated with poor tolerability [5]. This fact leads to frequent treatment discontinuation and therefore inadequate disease management; with a consequent poor effect in reducing the personal, social, and economic burden of migraine [5, 6].

In the last years, migraine-specific preventive treatments targeting the calcitonin-gene related peptide (CGRP) pathway have been approved [7–9]. Specifically, anti-CGRP monoclonal antibodies (MAbs) have a well-established effectiveness and tolerability [10], including compared to conventional oral preventive drugs [11] and therefore they represent a valuable option for migraine prevention [9]. However, their use in clinical practice is limited due to National Reimbursement policies which are driven by the apparent thought that they are expensive [12]. Specifically, in Spain, anti-CGRP MAbs can be prescribed after failure to three or more preventive treatments, one of them being onabotulinumtoxinA in case of chronic migraine.

Considering the need to reduce migraine burden for people and, in macroeconomic terms, for society, it is fundamental to understand the cost–benefit of anti-CGRP MAbs, especially in the actively working population, as it may help redefining the current migraine care and reimbursement policies.

Given that anti-CGRP MAbs are clinically effective already at short-term [13, 14], we aimed to evaluate their cost–benefit and work impact in a cohort of actively working patients with migraine at 3 and 6 months of treatment.

## Methods

This is a prospective study conducted in a Spanish headache clinic, between Feb 1st, 2020 and Jan 31st, 2023. We screened all consecutive migraine patients treated with anti-CGRP MAbs according to the Spanish national guidelines [7] and European Headache Federation (EHF) recommendations [8] (erenumab 140 mg monthly, fremanezumab 675 quarterly and galcanezumab 120 mg monthly + 240 mg loading dose). In Spain, National reimbursement policy allows for patients to be prescribed an anti-CGRP MAb when they suffer from at least 8 monthly migraine days (MMD) and have failed 3 or more migraine preventive drugs, being one of them onabotulinumtoxinA if chronic migraine (CM) [12]. To be included in the study participants had to be in the working age population group between 18 to 65 years old. Migraine Diagnosis was done according to the third edition of the International Classification of Headache Disorders criteria (ICHD-3) [15].

We assessed demographic data, medical history, migraine characteristics and preventive treatments as well as working conditions (part time/full time, number of hours/week) at an initial visit. We used electronic headache diaries (eDiary) to prospectively collect the monthly migraine days (MMD), monthly headache days (MHD) and monthly acute medication days (MAMD). Emergency room consultations during the study period were obtained from the health care resource utilization scale (HCRU). Before starting the treatment, each participant had completed at least one-month of baseline eDiary. Concomitant treatments were allowed if they were stable for at least one month before starting anti-CGRP MAbs. Patients were followed up every 3 months at an in-person visit, since the first administration of the anti-CGRP MAbs (M0). The work productivity and activity impairment questionnaire (WPAI) [16] was used to assess the employment status and was administered at each follow-up visit.

The primary outcome of this study was to assess the cost–benefit of anti-CGRP MAbs at 3 months (M3). Secondary outcomes were: 1) cost–benefit at 6 months, 2) changes in working status between M0 and M3, 3) cost–benefit at 3 months according to responder status 4) projected cost–benefit at one year per person. We defined as responders (RE) those patients with ≥ 50% reduction in MHD, partial responders (PR) between 30–49% reduction in MHD and non-responders (NR) < 30%.

For the cost–benefit analysis at 3 and 6 months, we used the variables reported in Supplementary Table 1. We calculated the cost–benefit as the difference of overall costs between M0 and M3 (or M6). Costs at each timepoint included direct and indirect costs.

For direct costs, we considered the prices of the anti-CGRP MAbs as medication notified prices from the Spanish National Drug Registry of the Ministry of Health [12]. Consultation costs related to follow-up (an outpatient visit every three months) were obtained from the Catalan Healthcare Institution [17]. Finally, using the MAMD and the number of emergency visits from the HCRU, we calculated the costs related to acute medication use and healthcare resource utilization [18, 19].

For indirect costs, data from the WPAI questionnaire were used to calculate work time loss (absenteeism) and work impairment (presenteeism). Using the average hourly salary published by the Institute of statistics of Catalonia [20] we calculated the indirect working costs attributed to headache at M3 and M6. Absenteeism cost (€/month) = Average hourly salary *percentage of work time loss due to headache (time lost/(time lost + time worked)) *8(work hours/day) *5(workdays/week) *4(weeks/month). Presenteeism cost (€/month) = Average hourly salary * time worked excluding absenteeism ((work time – work time loss)/work time) *Productivity impairment (from WPAI) *8(work hours/day) *5(workdays/week) *4(weeks/month) [21]. The indirect costs related to time spent to get to the hospital, visits at the clinic and drug administration were considered negligible.

Furthermore, recent studies have reported that anti-CGRP MAbs are effective at longer term in real world [22]. Based on this assumption, we projected the savings achieved after 3–6 months of treatment over a year.

### Statistical analysis

Nominal variables, including sex, diagnosis, the presence of aura, the type of anti-CGRP MAb prescribed, and the emergency visits are presented as frequencies and percentages. Conversely, quantitative variables like age, HDM, MDM, MAMD are described using mean and standard deviation. Additionally, the total working hours, absenteeism and presentism are reported by the mean percentage present in our cohort.

We analysed the longitudinal variances with a 3-month and 6-month interval. Differences in MHD, MMD, MAMD, absenteeism and presenteeism over time (M0-M3; M0-M6) were compared using a paired Wilcoxon signed-rank test, after checking their distribution. In this way each patient constituted his own control. All patients including those who discontinued were included in the cost analysis.

Considering the simultaneous analysis of multiple statistical tests, we used the False Discovery Rate (FDR) method to adjust the *p*-values. Our significance threshold for determining statistical significance was set at *p* < 0.05 after applying this adjustment. Due to the exploratory nature of our research and the limited available data, no statistical power analysis was conducted prior to the analysis and the sample size was determined based on the available data.

Statistical analysis was performed using the version 4.2.2 of R software and figures were produced using the package *ggplot2*.

## Results

### Characteristics of the working-age cohort

From 470 patients treated with anti-CGRP MAbs, 426 were at working age. Of these, 256 participants had complete data, the 57.8% (148/256) were employed at both M0 and M3. Reasons for not working at M0 (96/256) were: 53% unemployed (*n* = 51, from which a 55% (*n* = 28) reported migraine as the main reason for this condition), 29.1% homemakers (*n* = 28), 8.3% students (*n* = 8) and 9.4% unknown (*n* = 9). If we consider patients who could potentially be employed (patients in working age without students and homemakers), the unemployment rate in our cohort stands at 24.5% (51/208). Reasons for not working are graphically represented in the Supplementary Fig. 1.

**Fig. 1:**
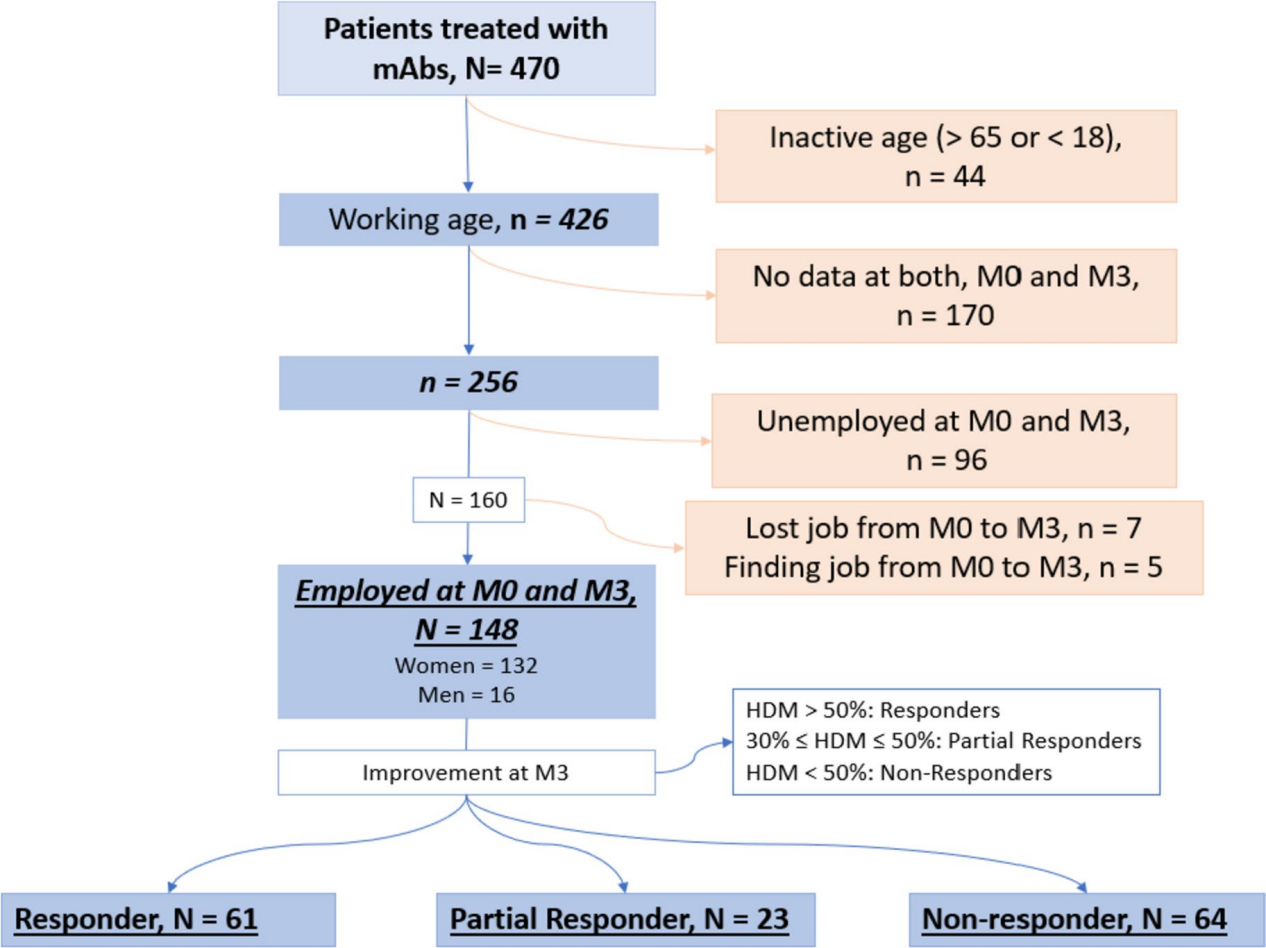
Flowchart of study participants. From 470 patients treated with anti-CGRP MAbs, 426 were at working age. Of these, 256 participants had complete data, of which 57.8% (148/256) were employed at both M0 and M3

From the final cohort of employed participants, 89.2% (132/148) were women and mean age was 48.0 ± 8.5 years. Sixty-two-point two percent (92/148) had CM. Figure 1 shows the participants flowchart, whereas Table 1 cohort baseline characteristics.

**Table 1:** Baseline characteristics of the study cohort (*n* = 148)

Variables at baseline	**Total**, *N* = 148
**Age**, mean (SD)	47.6 (8.5)
**Sex** (woman), n (%)	132 (89.2%)
**Years of evolution**, mean (SD)	28.3 (12.3)
**Diagnosis**, n (%)	
Episodic migraine	56 (37.8%)
Chronic migraine	92 (62.2%)
**Anti-CGRP**, n (%)	
Erenumab	93 (62.8%)
Galcanezumab	34 (23%)
Fremanezumab	21 (14.2%)
**Total working hours**, mean (SD)	37.1 (8.2)
**Absenteeism**, median (Q1-Q3)	0 (12.3–0)
**Presentism**, median (Q1-Q3)	40 (66.3–0)

*Abbreviations: SD Standard deviation, Q1-Q3 Interquartile range*

### Efficacy results and follow-up results

At 3 months, a statistically significant one-week reduction in headache days/month was found (M0: 17.8 ± 7.4 vs. M3: 10.9 ± 7.7; *p* < 0.001) and improvement in acute medication days/month (M0: 12.2 ± 6.3 vs. M3: 7.3 ± 5.0; *p* < 0.001) (Table 2). Seventeen patients discontinued anti-CGRP MAbs at M3: 13 (76.5%) for lack of efficacy, 1 (5.9%) for lack of tolerability and 3 (17.6%) for other reasons.

**Table 2:** Comparison between M0 (baseline) and M3 (3 months after treatment with MAbs)

	M0	M3	Difference	*p*-value
**Headache days/month**, mean (SD)	17.8 (7.4)	10.9 (7.7)	-6.9	** < 0.001**
**Migraine days/month**, mean (SD)	12.7 (7.1)	6.4 (6.1)	-6.3	** < 0.001**
**Acute medication days/month**, mean (SD)	12.2 (6.3)	7.3 (5)	-4.9	** < 0.001**
**Triptans used/month**, mean (SD)	8.4 (6.6)	4.2 (4.6)	-4.2	** < 0.001**
**MIDAS score**, median (Q1-Q3)	50 (95.0–27.8)	14 (45.3–4.0)	-36	** < 0.001**
**HIT-6 score**, median (Q1-Q3)	65 (69.0–61.8)	55.5 (62–48.0)	-9.5	** < 0.001**
**Total working hours**, mean (SD)	37.1 (8.2)	36.5 (8.8)	-0.6	0.347
**Absenteeism** (%), mean (SD)	13.4 (25.8)	8.4 (21.9)	-5	** < 0.001**
**Presenteeism** (%), mean (SD)	42.7 (29.5)	24.3 (27)	-18.4	** < 0.001**

*Absenteeism: % of hours not worked due to headache. Presenteeism:* laboral productivity affected by headache*. Abbreviations: SD* Standard deviation*, Q1-Q3 Interquartile range, WPAI* Work productivity and activity impairment*, HIT-6 score* Headache impact severity level*, MIDAS* Migraine Disability Assessment

Patients who persisted with the treatment at month 6 demonstrated a sustained improvement in the analysed response parameters: headache days/month, acute medication days/month, absenteeism and presenteeism (Supplementary Table 2).

### Cost–benefit analysis

**Fig. 2:**
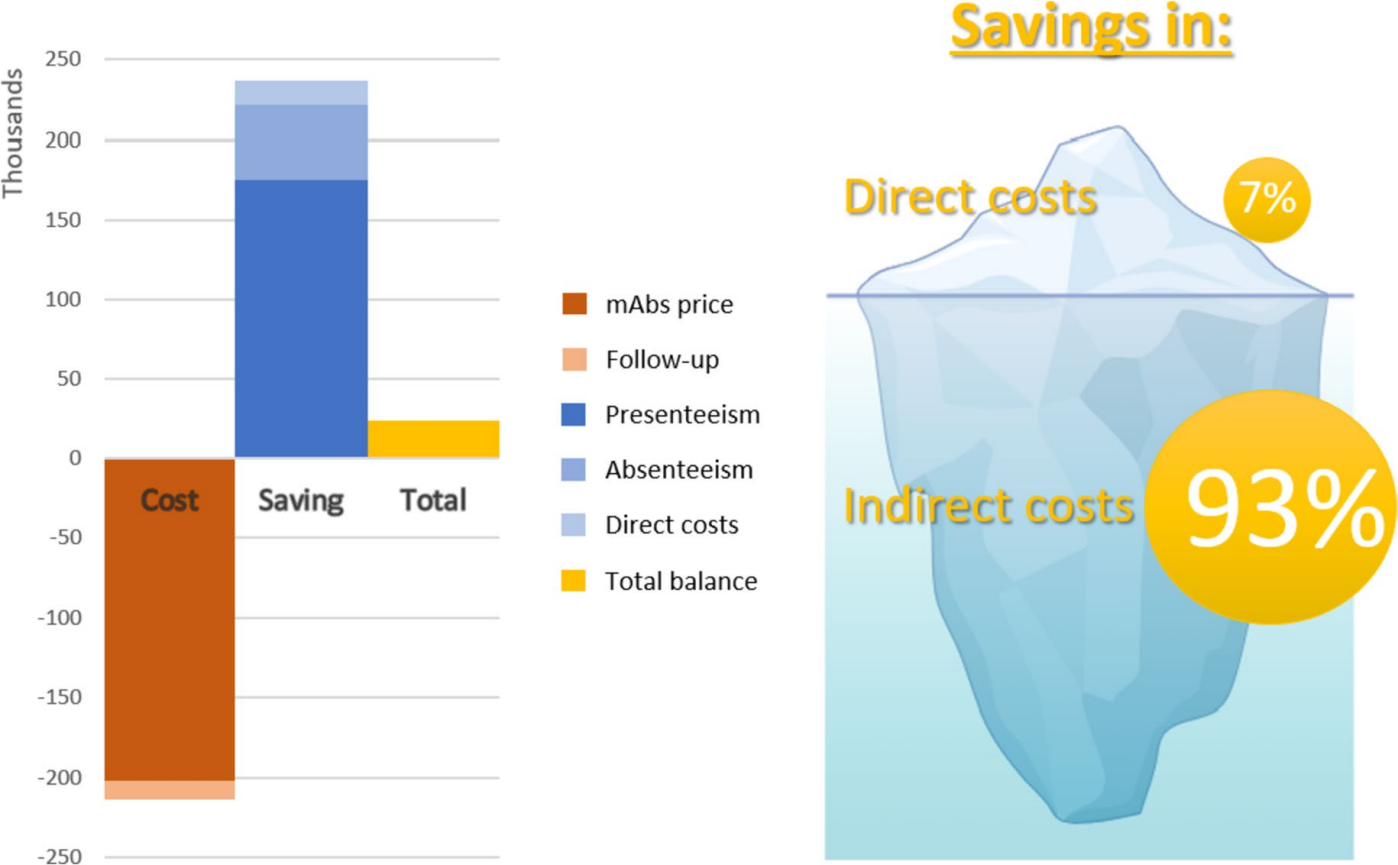
Graphical representation of the economic balance and the relative importance of the savings in indirect costs in comparison with the savings in direct costs derived from the treatment with anti-CGRP MAbs. The main cost of these therapies are in terms of direct expenses (the drugs costs). However, the 93% of the savings are indirect (reduction in absenteeism and presenteeism). This makes the benefit that they socially produce in economic terms not so evident in the first instance in comparison to their costs, but not less important. *Image generated using BioRender*

In relation to the work impact, we observed approximately a 40% reduction in work impact variables at 3 months (37% reduction in absenteeism, M0: 13.4% vs. M3 8.4%; *p* = 0.001; and 43% reduction in presenteeism, M0: 42.7% vs. M3: 24.3%; *p* < 0.001). Consultations at the emergency room (ER) decreased by 55.9% (M0: 47.7 emergency visits/month vs. M3: 21 emergency visits/month; *p* < 0.001) (Table 2). Figure 2 and Table 3 show all direct and indirect costs at M0 and M3 as well as savings after anti-CGRP treatment per patient. The improvement in absenteeism and in presenteeism allowed a mean saving of 105.4 euros/month per patient and 394.3 euros/month per patient, respectively, with an overall 3-month saving in indirect costs of 1499.1 euros/patient. Savings in direct costs, due to reduction in acute treatment use and ER visits, accounted for another 33.9 euros/month per patient. Thus, savings after 3 months of anti-CGRP treatment reached a mean of 1600.8 euros/patient. However, considering that the costs of anti-CGRP MAbs for 3 months of treatment are 1361.0 euros/patient (453.67 × 3) and that of the outpatient visits are 80 euros/patient, the cost-saving analysis found a final mean saving of 159.8 euros/patient for the firsts 3 months of treatment. For the overall cohort, the savings reached 23,650.4 euros in the first 3 months (Table 3).

**Table 3:** Costs and savings at 3 months per patient

		**M0** *(EUR)*	**M3** *(EUR)*	**Monthly balance per patient** *(EUR)*	**Total balance** *(EUR per patient for 3 months of treatment)*
**Direct Costs**	anti-CGRP MAbs	0.0	453.67	-453.67	**-1361.0**
	Follow-up (1 outpatient visit)^a^	0.0	80	-26.7	**-80**
**Direct Savings**	Emergency visits	55.6	24.5	+ 31.1	** + 93.3**
	Triptans	5.4	2.6	+ 2.8	** + 8.4**
**Indirect Savings**	Absenteeism	330.5	225.1	+ 105.4	** + 316.2**
	Presenteeism	929.7	535.4	+ 394.3	** + 1182.9**
				**Total per patient**	** + 159.8**
				**TOTAL**	** + 23,650.4**

^a^Patients treated with MAbs received an additional outpatient visit every 3 months during the follow-up, which was encountered in the cost–benefit analysis. The rest of clinical visits were the same as patients with migraine and without this treatment

At month 6, applying the same analysis to patients who continued anti-CGRP MAbs and had data available in all M0, M3 and M6 (*n* = 83), we observed an increase in savings at month 6 of 26.7 euros/month in indirect costs compared to month 3. There was also an increase in savings of 24.9 euros/month in direct cost. The total increase in savings reported at month 6 was 159.9 euros/patient compared to month 3. Which added to the previously calculated savings sums up to a total of 314.7 euros per patient during the second trimester, and an overall saving of 26,120.1 euros. Supplementary Table 3 reports all costs and savings at M3 and M6.

After observing that savings calculated for month 3 remained stable in month 6, we estimated the annual savings per patient after a year of treatment to be 639.2 euros.

### Cost–benefit according to responder status at 3 months

The patients were classified according to the reduction in MHD in three groups: 41.2% (61/148) responders (> 50% reduction); 15.5% partial responders (30–50% reduction) and 43.2% (64/148) non-responders (< 30% reduction) (baseline characteristics according to response rate are shown in Supplementary Table 4).

After 3 months of treatment, partial responders (*n* = 23) showed a statistically significant reduction in absenteeism (M0 18.8% vs. M3 6.8%; *p* = 0.017) and presenteeism (M0 39.6% vs. M3 22.2%; *p* = 0.017). Non-responders (*n* = 64) showed significant reduction in presenteeism (M0 49.6% vs. M3 37.0%; *p* = 0.01) but not absenteeism (M0 16.0% vs. M3 12.9%; *p* = 0.343). In terms of cost-savings, partial responders saved 488 euros/patient whereas non-responders did not (-501.9 euros/patient) (Supplementary Tables 5 and 6).

### Changes in work status

Of the 256 patients, 17 were students and homemakers. Therefore, 239 were the patients susceptible of changing employment status. The 94.5% (226/256) of the patients did not present changes in their employment status three months after starting the treatment. From the 61/256 unemployed patients, five patients got employed by month 3: 40% (2/5) reported the improvement in their migraine as the main reason for this change, 40% (2/5) for other reasons and in one case it was unknown.

Of the 256 patients, 60.5% (155/256) were employed at the beginning of the study. Of them, seven changed their status to unemployed by month 3. The reasons were 14.3% (1/7) migraine, 71% (5/7) other causes and 14.3% (1/7) unknown. The 57% (4/7) were responders and 43% (3/7) were non-responders.

## Discussion

The working-age population represents the main target for migraine care because it is the most affected in terms of disease incidence and prevalence [1, 23, 24]. Illness during working ages had a great economic impact on society [24]. Our study is the first one to analyse in a prospective way the impact of starting anti-CGRP MAbs in working-age migraine patients to evaluate the cost–benefit of these new, and apparently more expensive, migraine-specific preventive treatments. These are our findings:

First, in patients actively working, anti-CGRP MAbs are cost-effective already at 3 months, mainly because the drug costs are compensated by the savings obtained by the reduction in absenteeism and presenteeism. Additionally, we observed a stability of this effect in patients who maintained the treatment up to 6 months and our projected annual cost-analysis also could lead to long-term savings. Other studies, using specific models based on clinical trials outcomes, have estimated that anti-CGRP drugs are in general cost-effective [25–28]. Our study, based on real-world data, has a direct approach on how we measure work-related costs and supports those estimations at least in the group of patients that are employed. Our findings provide evidence that the current criteria for reimbursement and prescription in Spain, requiring 3 previous treatment failures, are no longer supported by economic reasons. Instead, we propose that anti-CGRP MAbs should be considered as an earlier line of treatment, as also recommended by the EHF [8].

Second, a significant proportion of our patients treated with anti-CGRP MAbs, around 24.5%, (51/208) that could potentially work, are unemployed. One of the factors that could contribute to this working status is the difficult-to-treat migraine they suffer, and have suffered during their life, where they had to study and position themselves professionally while having migraine, and currently have. This finding reflects the fact that the population fulfilling criteria for anti-CGRP MAbs is treatment-resistant [29]. Interestingly, whether they respond or not, the working status does not seem to change. This could either be due to the short timepoint of our study (3 months) or to personal, social and community factors that prevent work reintegration after being undertreated for migraine for a long time. Considering these findings, it would be interesting to assess in the future whether treating patients earlier may avoid a prolonged unemployment status caused by the disease, with a beneficial impact not only in reducing the personal migraine burden but also potential societal costs related to loss of productivity.

Third, the overall treatment benefit is able to compensate the costs for those who are non-responders. We analysed the baseline characteristics of non-responders’, finding no statistically significant differences when compared to rest of the cohort. Since there are no predictors of response at present [30, 31], our study provides the evidence that all patients can and should be treated. Moreover, the economic impact of people discontinuing the treatment is higher initially, but it will be mitigated by the time as only those patients who respond to anti-CGRP MAbs will continue the treatment. This fact may also contribute to underestimating the potential savings achievable at a longer-term, since the follow up of our study is limited to 6 months. Thus, our annual projection of savings may also be underestimated.

Finally, we observed that partial responders significantly improve as well in terms of absenteeism and presenteeism. As these variables are the main determinants for the treatment-related savings, even in < 50% responders who are actively working, anti-CGRP MAbs could be cost-effective. A 50%-threshold that qualifies non-responders should probably considered no longer representative from both the clinical and economic perspective and lowering the threshold to 30%, at least in real-world, should be considered.

Overall, our study, coupling precisely collected real-world data with economic evaluations, demonstrates that treatment with anti-CGRP MAbs is sustainable in the actively working migraine population and should be offered earlier. Although our study was only carried out on working patients, we believe that the treatment should be equally available to unemployed patients, since economics is just one facet of the potential benefits that treatment with MAbs provides.

The present study is not exempt of limitations:

First, it has been conducted in a specialized headache clinic and a selection bias may have occurred, possibly including more severe and resistant patients but with greater potential for improvement. So, our results cannot be generalized to low-frequency episodic migraine.

Second, as we used WPAI for the economic assessment, we were able to estimate the cost–benefit only for those patients actively working, and future studies should have to determine with proper real-data if the treatment is cost-effective in the overall population, including people unemployed or not in working age. Additionally, despite the WPAI scale has been validated in migraine, it remains a self-reported scale, introducing subjectivity especially when assessing productivity. Nevertheless, this potential bias is mitigated as we are evaluating changes of WPAI over time (paired samples), where each individual serves as his own control.

Another limitation is that a non-negligible proportion of patients had to be excluded from the study due to incomplete WPAI data at M0 or M3, rendering their inclusion in the analysis impossible. We have analysed the response rates of these patients and have not identified significant variations when compared to the rest of the cohort (Supplementary Table 7). Although the exclusion of these patients constitutes a potential limitation of the study, there is no evidence to suggest that the final analysed cohort is not a representative sample.

Finally, because of the economic evaluation, our results are applicable only in our country, but we urge the need of replicating these assessments worldwide.

## Conclusions

In our real-world study, anti-CGRP MAbs reduce absenteeism and presenteeism in people actively working, with related savings that overcome the costs of the drug. Considering that nowadays their cost is the main determinant that limits and delays their prescription, our results open up the possibility of earlier prescription of these treatments for migraine, as in this population they are economically sustainable.

### Supplementary Information

**Additional file 1.**

## Data Availability

All data are available and any anonymized data will be shared by request from any qualified investigator.

## Abbreviations

CGRP: Calcitonin gene-related peptide
MAbs: Monoclonal antibodies
ER: Emergency room
GBD: Global burden disease
DALYs: Disability-adjusted life years
eDiary: Electronic headache diaries
EHF: European headache federation
ICHD-3: International Classification of Headache Disorders, 3rd edition
HCRU: Health care resource utilization scale
WPAI: Work productivity and activity impairment
CM: Chronic migraine
EM: Episodic migraine
M0: Baseline
M3: Time at 3 months
M6: Time at 6 months
MHD: Monthly headache days
MMD: Monthly migraine days
MAMD: Monthly acute medication days
RE: Responders (> 50% reduction in monthly headache days)
PR: Partial responders (30–49% reduction in monthly headache days)
NR: Non-responders (< 30% reduction in monthly headache days)
SD: Standard deviation
Q1-Q3: Interquartile range
