# Supplementary material for: Early and annual projected savings from anti-CGRP monoclonal antibodies in migraine prevention: a cost-benefit analysis in the working-age population
Source: J Headache Pain. 2024 Feb 12;25(1):21. doi: 10.1186/s10194-024-01727-0 (PMC10860274; doi:10.1186/s10194-024-01727-0)
Supplement: Supplementary file 1 — Additional file 1. [file 10194_2024_1727_MOESM1_ESM.docx]

Supplementary Material

***Supplementary Table 1.- Variables and costs used for the cost-benefit analysis.*** *Drug prices have been obtained from the Spanish national records of the Ministry of Health* (1,2)*, costs related to hospital care from the reports of the Catalan Health Institute* (3) *and the most recently published hourly salary by the institute of statistics of Catalonia* (4)*.*

|  |  |  | **Cost per unity** (euros) | **Period** |
| --- | --- | --- | --- | --- |
| **Direct costs** | Anti-CGRP MAbs | Erenumab | 445 | Monthly |
|  |  | Galcanezumab | 450 |  |
|  |  | Fremanezumab | 498 |  |
|  | Follow-up | Outpatient visit | 80 | Every 3 months |
|  | Emergency visits | | 172.5 | On demand |
|  | Triptans | | 1.95 | On demand |
| **Indirect Costs** | Hourly salary | | 17.93 |  |

***Supplementary Table 2.- Comparison of the results variables at time M0, M3 and M6 for the 83 patients who completed the follow up.*** *We can appreciate great improvement between M0 and M3, maintaining it in month 6. No statistically significant changes were identified in the Wilcoxon test between month 3 and month 6. Abbreviations: M0 = Baseline; M3 = after 3 months of treatment; M6 = after 6 months of treatment*

| n=83 | M0 | M3 | M6 | p-value  M3 vs M6 |
| --- | --- | --- | --- | --- |
| **Headache days/month**, mean (SD) | 18.4 (7.6) | 11 (8) | 10.5 (8.3) | 0.74 |
| **Migraine days/month**, mean (SD) | 13.2 (7.3) | 6.3 (6.2) | 5.5 (6.2) | 0.55 |
| **Acute medication days/month**, mean (SD) | 12.1 (6) | 7.2 (4.9) | 7.2 (5.7) | 0.76 |
| **Triptans used/month**, mean (SD) | 8.3 (6.7) | 4.1 (4.7) | 4.2 (4.9) | 0.78 |
| **Absenteeism** (%), mean (SD) | 12.7 (26.2) | 8.8 (25.6) | 10.3 (24.6) | 0.39 |
| **Presenteeism** (%), mean (SD) | 41.8 (30.3) | 21.2 (26.6) | 20.5 (33) | 0.55 |

***Supplementary Table 3.- Cost-benefit balance at months 3 and 6.*** *The table shows comparisons between month 0 and month 3 for 148 patients; and between month 3 and month 6 for 83 patients. Abbreviations: M0 = Baseline; M3 = after 3 months of treatment; M6 = after 6 months of treatment; EUR = euros; MAbs = Monoclonal antibodies*

|  |  | M0  (EUR per month and patient) | M3  (EUR per month and patient) | Monthly balance  M0 vs. M3 | Total balance  M0 vs. M3  (n=148) | M3  (EUR per month and patient) | M6  (EUR per month and patient) | Monthly balance  M3 vs. M6 | Total balance M3 vs. M6  (n=83) |
| --- | --- | --- | --- | --- | --- | --- | --- | --- | --- |
| Direct cost | Anti-CGRP MAbs | 0 | 453.67 | -453.67 | -1361 | 453.67 | 453.67 | 0 | 0 |
|  | Follow-up | 0 | 80 | -26.6 | -80 | 80 | 80 | 0 | 0 |
| Direct Savings | Emergency visits | 55.6 | 24.5 | +31.1 | +93.3 | 27 | 2.1 | +24.9 | +74.7 |
|  | Triptans | 5.4 | 2.6 | +2.8 | +8.4 | 2.5 | 2.5 | 0 | 0 |
| Indirect Savings | Absenteeism | 330.5 | 225.1 | +105.4 | +316.2 | 238.6 | 220.2 | +18.4 | +55.2 |
|  | Presenteeism | 929.7 | 535.4 | +394.3 | +1182.9 | 455.2 | 446.9 | +8.3 | +25 |
|  |  |  |  |  | 159.8/patient |  |  |  | Additional 154.9/patient |
|  |  |  |  |  | Total= 23650.4 |  |  |  | Additional total= 12856.7 |

***Supplementary Table 4.- Comparison between the baseline characteristics of responders (>50% reduction in monthly headache days) vs. partial responder’s and non-responders’ (<50% reduction in monthly headache days).*** *Abbreviations: SD = Standard deviation; Q1-Q3 = Interquartile range; WPAI = Work productivity and activity impairment*

| Variables at baseline | **Total**,  N =148 | **Responders**,  n=61 | **Partial responders’ and non-responders**, n=87 | **p-value** |
| --- | --- | --- | --- | --- |
| **Age**, mean (SD) | 47.6 (8.5) | 47.9 (7.8) | 47.4 (9.1) | 0.743 |
| **Sex** (woman), n (%) | 132 (89.2%) | 54 (88.5%) | 78 (89.7%) | >0.999 |
| **Years of evolution**, mean (SD) | 28.3 (12.3) | 30.1 (10.5) | 27.1 (13.3) | 0.163 |
| **Diagnosis**, n (%)  Episodic migraine  Chronic migraine | 56 (37.8%)  92 (62.2%) | 21 (34.4%)  40 (65.5%) | 35 (40.2%)  52 (59.8%) | 0.48 |
| **Anti-CGRP**, n (%)  Erenumab  Galcanezumab  Fremanezumab | 93 (62.8%)  34 (23%)  21 (14.2%) | 37 (60.7%)  13 (21.3%)  11 (18%) | 56 (54.4%)  21 (24.1%)  10 (11.5%) | 0.575 |
| **Total working hours**, mean (SD) | 37.1 (8.2) | 38 (8.6) | 36.4 (7.9) | 0.257 |
| **Absenteeism**, median (Q1-Q3) | 0 (12.3-0) | 0 (10.8-0) | 0 (13.4-0) | 0.073 |
| **Presentism**, median (Q1-Q3) | 40 (66.3-0) | 40 (60-10) | 50 (70-20) | **0.036** |

***Supplementary Table 5****. –* ***Costs and savings in euros for partial responders (30-50%) at month 3, n=23.*** *Abbreviations: M0 = Baseline; M3 = after 3 months of treatment; MAbs = Monoclonal antibodies*

|  |  | M0  (per patient) | M3  (per patient) | Monthly balance | Total balance  (per patient) |
| --- | --- | --- | --- | --- | --- |
| Direct cost | Anti-CGRP MAbs | 0 | 453.7 | -453.7 | -1361 |
|  | Follow-up | 0 | 80 | -26.6 | -80 |
| Direct savings | Emergency visit | 25 | 2.5 | +22.5 | +67.5 |
|  | Triptans | 6.2 | 3.1 | +3.1 | +9.2 |
| Indirect savings | Absenteeism | 505.4 | 192.7 | +312.6 | +937.8 |
|  | Presenteeism | 834.1 | 529.2 | +304.8 | +914.5 |
|  |  |  |  |  | 488 |
|  |  |  |  |  | Cohort’s total=11224 |

***Supplementary Table 6. –*** ***Costs and savings in euros for non-responders (<30%) at month 3, n=64****. Abbreviations: M0 = Baseline; M3 = after 3 months of treatment; MAbs = Monoclonal antibodies*

|  |  | M0  (per patient) | M3  (per patient) | Monthly balance | Total balance  (per patient) |
| --- | --- | --- | --- | --- | --- |
| Direct cost | Anti-CGRP MAbs | 0 | 453.7 | -453.7 | -1361 |
|  | Follow-up | 0 | 80 | -26.6 | -80 |
| Direct savings | Emergency visit | 66.5 | 46.7 | +19.8 | +59.3 |
|  | Triptans | 5.1 | 3.8 | +1.3 | +3.9 |
| Indirect savings | Absenteeism | 401.7 | 362.8 | +39 | +116.9 |
|  | Presenteeism | 997.8 | 744.8 | +253 | +759 |
|  |  |  |  |  | -501,9 |
|  |  |  |  |  | Cohort’s total=-32121.6 |

***Supplementary Table 7.- Comparison between the responder rates of the 170 patients excluded from the study due to lack of WPAI data and the final analysed cohort (n=148).***

|  | **Final analysed cohort**  n = 148 | **Excluded patients due to not available WPAI**  n = 170 |
| --- | --- | --- |
| **Responders** (>50% decrease in monthly headache days) | 61/148 (41.2%) | 66/170 (38.8%) |
| **Partial responders** (30-49% decrease in monthly headache days) | 23/148 (15.5%) | 18/170 (10.6%) |
| **Non-responders** (<30% decrease in monthly headache days) | 64/148 (43.2%) | 82/170 (48.2%) |
| No available frequency data | 0/148 (0%) | 4/170 (2.4%) |

***Supplementary Figure 1. Reasons for not being actively working at the time the study was conducted, reported by the 96 patients.*** *53% were unemployed (n=51, from which a 55% (n=28) reported migraine as the main reason for this condition), 29.1% homemakers (n=28), 8.3% students (n=8) and 9.3% unknown (n=9).*

***
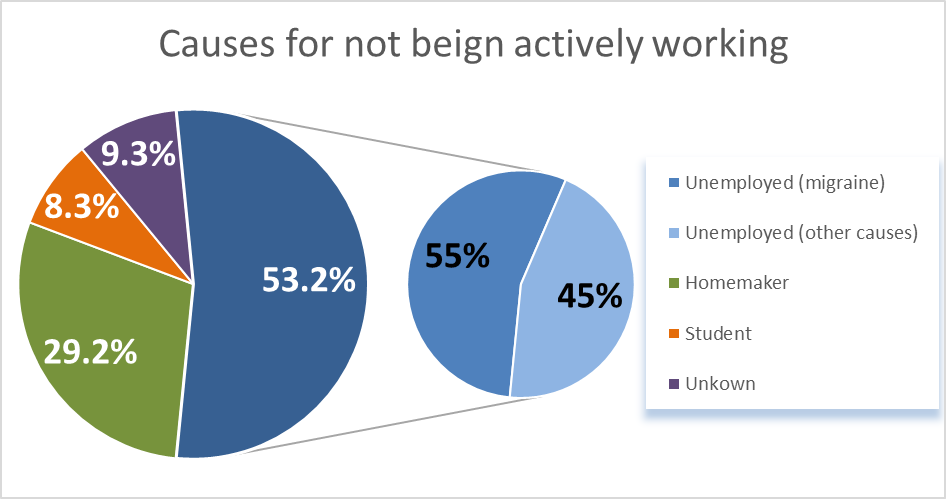
***

***Supplementary Figure 2. Triptans used/month, absenteeism and presenteeism compared between baseline (M0) and month 3 (M3).*** *They are separately compared for non-responders, partial-responders and responders.*


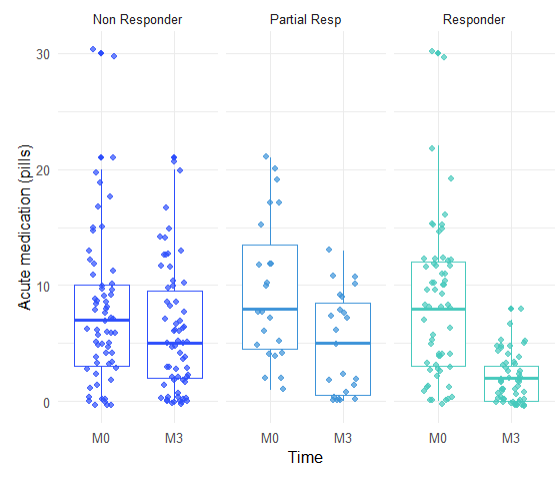


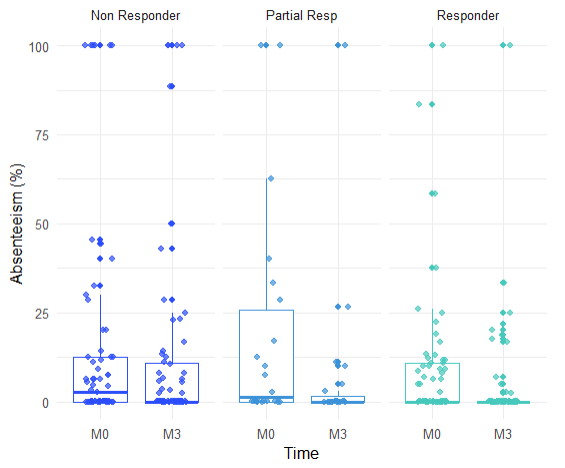


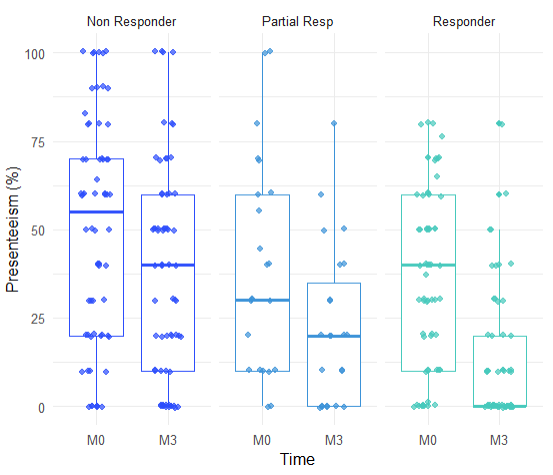


**Bibliography**

1. Ministerio de sanidad de España. Acuerdos de la comisión interministerial de precios de los medicamentos. 2023.

2. Ministerio de Sanidad de España. Listado de agrupaciones homogéneas de medicamentos y precios menores - Octubre 2023. 2023.

3. de Salut D. ORDRE SLT/71/2020, de 2 de juny, per la qual es regulen els supòsits i. 2020 [cited 2023 Apr 2]; Available from: https://www.gencat.cat/dogc

4. Institut d’Estadística de catalunya. https://www.idescat.cat/indicadors/?id=anuals&n=10402. 2023. Salari brut anual i guany per hora. Per sexe i sectors d’activitat.
